# Enhancing student satisfaction via social comparisons

**DOI:** 10.1371/journal.pone.0335910

**Published:** 2025-11-07

**Authors:** Yumei Mu, Julian Givi, Stephen He

**Affiliations:** 1 Davis College of Business and Economics, Radford University, Radford, Virginia, United States of America; 2 John Chambers College of Business and Economics, West Virginia University, Morgantown, West Virginia, United States of America; 3 Carlos Alvarez College of Business, The University of Texas at San Antonio, San Antonio, Texas, United States of America; Bar-Ilan University, ISRAEL

## Abstract

University tuition and fees impact student engagement and school enrollment. Thus, it is important that students be satisfied with what they are paying. This research incorporates social comparisons in communications to students and examines the impact that these comparisons have on student satisfaction with their cost of attendance and overall university experience. In particular, this work employs an experimental design with two conditions. In the *Increase* condition, participants learned that a counterpart university planned to increase its tuition and fees, while their own university kept the cost of attendance constant; in the *Constant* condition, participants learned that both the counterpart university and their own kept tuition and fees constant. The dependent variables were participants’ satisfaction with their own university’s tuition and fees, as well as their overall satisfaction with their university experience. In addition, this research examined relative value as a potential mediating variable influencing these outcomes. The results demonstrate that students become relatively more satisfied when their tuition and fees remain constant, but a counterpart university increases its tuition and fees (the *Increase* condition) versus keeps these costs constant (the *Constant* condition). In other words, simply knowing that another university has raised prices (vs. kept them constant) but theirs has not, enhances student satisfaction with tuition and fees, which leads to greater satisfaction with their overall university experience. These findings emerge because students perceive the price they pay for their cost of attendance to be of higher relative value when another university raises prices (vs. keeps them constant). Notably, whereas most research demonstrates how consumer satisfaction can be helped by an organization spending money, this research identifies a cost-free method that universities can use to enhance student satisfaction.

## Introduction

In February 2024, Dr. Ruth Gottesman made a generous donation of $1 billion to the Albert Einstein College of Medicine, enabling many current and future students to pursue their education tuition-free [1]. This philanthropic gesture was made to not only help the college’s students but also stimulate an increase in applications and enrollment. However, many students across the United States are not lucky enough to have their tuition burdens lifted. In fact, the cost of higher education, including tuition and fees, has been steadily increasing over recent decades [2,3]. The financial strain of education has become increasingly burdensome for many students and their families, often resulting in substantial debts upon graduation [4]. Such financial constraints have even dissuaded some individuals from pursuing university education altogether or from returning due to the prohibitive costs [5,6].

In recent years, exacerbated by the impact of the pandemic and a decrease in the eligible population, these high costs have contributed to a sharp decline in university enrollments [7]. This trend is consistent with research findings indicating that high costs negatively affect student enrollment, retention, satisfaction, and experience [8–10]. Nevertheless, some universities have opted to refrain from increasing their tuition and fees for several years [11]. This raises an intriguing question: Do students at such universities experience relatively higher satisfaction, given that their own cost of attendance is remaining constant, while the cost of attendance for other students is increasing? The present research seeks to address this question.

This research investigates how a consumer’s satisfaction with their payment and consumption experience is affected when the price (or cost) they pay remains constant but the costs for their peers are increased (the *Increase* condition) versus when they are not (the *Constant* condition). This research utilizes university education as the consumption context and introduces social comparisons as a key component in the communication regarding tuition and fees. When a student’s tuition and fees at their own university remain unchanged for two consecutive years, while a counterpart university raises its prices in the second year (vs. keeps these costs constant), it is anticipated that students become more satisfied with the price they pay and consequently their overall university experience. Moreover, it is hypothesized that this occurs because students perceive the price they pay for their tuition and fees to be of higher relative value when another university raises prices.

This research makes both theoretical and practical contributions. Theoretically, this work demonstrates that even if the costs consumers pay and the benefits they receive remain constant, their satisfaction can increase due to the relatively higher value for their payment when they learn that fellow consumers have (vs. have not) had their costs increased. This finding adds to the robust literature on social comparisons in the consumption context [12]. From a practical standpoint, this research offers a strategy to enhance consumer satisfaction without requiring additional investments in the provided offerings (i.e., by communicating that other consumers are paying more). Typically, service providers need to invest more resources to enhance customer satisfaction. Therefore, this research identifies an important cost-saving opportunity for service providers. Considering the pivotal roles that price plays in the marketing mix [13] and consumer decisions [14], the findings provide important guidance to marketers in their marketing communication efforts.

## Theoretical development

Higher education costs vary globally, from free education in countries like Norway, Finland, and Germany [15], to fee-based systems in countries like the United States, Canada, and Japan [16]. In the United States, student loans are prevalent. For example, in 2020–2021, over half of graduates finished their undergraduate programs with loans [17]. This underscores the important role that tuition and fees play when students choose which university to attend [18]. Tuition and fees often increase over time, driven by factors such as inflation, service expansion, rising labor costs, and workforce growth [19,20]. Nonetheless, some universities choose to maintain their tuition and fees at a constant level for a few years, often due to increased funding from outside sources [20,21].

Previous research has examined the impact of tuition increases on student enrollment, engagement, and graduation rates. For example, tuition increases have been shown to reduce enrollment [9]. Additionally, retention rates tend to decline as tuition increases [10]. However, when tuition increases are anticipated rather than sudden, they may motivate students to complete their degrees more quickly, resulting in higher on-time graduation rates [22]. Despite the considerable attention given to tuition increases, relatively little research has examined student behavior when their cost of attendance remains constant.

It is clear that students tend to be more satisfied with the price they pay for tuition and fees when their cost of attendance remains constant rather than increases. However, maintaining satisfaction becomes a challenge for a university when it holds its tuition and fees constant over the years, as this benefit may gradually be taken for granted [23,24]. How can universities sustain student satisfaction with costs in such circumstances? This research aims to explore how university communication strategies may address this challenge. Specifically, it investigates how students’ levels of satisfaction with their cost of attendance are influenced when the tuition and fees at their university remain constant over two years while the tuition and fees at a counterpart university increase in the second year (vs. also remain constant over two years). Many universities provide information about the cost of attendance at both their university as well as others. For example, the State University System of Florida [25] annually tracks the cost of attendance at each member institution, enabling students to compare costs directly (see Appendix 1). The present work suggests that when students learn that the costs at a counterpart university increase while their own costs remain unchanged, they will exhibit relatively higher satisfaction with the price they pay for tuition and fees.

Moreover, increased student satisfaction likely extends beyond tuition and fee satisfaction to one’s satisfaction with their overall university experience. Consumer attitudes toward costs are closely linked to satisfaction with the broader consumption experience. When consumers perceive good value in a transaction, they are more likely to be satisfied with both the payment and the experience [26]. This concept is equally relevant in higher education. Students who are satisfied with what they are charged in tuition and fees will tend to view their investment more favorably, enhancing their overall satisfaction with the university experience [27]. In sum, our first hypothesis is as follows:

H1: Students become relatively more satisfied with (a) their tuition and fees and (b) their overall university experience when they learn that their tuition and fees remain constant, but a counterpart university increases its tuition and fees (vs. keeps these costs constant).

### Social comparisons

The prior hypothesis is rooted in the psychological concept of social comparisons, whereby consumers engage in self-evaluations by comparing themselves to similar others on various dimensions, including material possessions, social status, and other aspects [28]. Even when a consumer’s personal situation remains unchanged, their cognitive assessments and emotional responses often vary based on whether their relative standing is superior or inferior to that of their peers on a specific criterion [12,29]. When their standing is superior, consumers tend to engage in downward comparisons and typically experience positive emotions [30]. For example, in these situations, consumers often experience heightened positive affect [31], increased subjective well-being [32], and a sense of superiority [33]. Conversely, when their standing is inferior, consumers tend to engage in upward comparisons and typically experience negative emotions [34]. For example, in such situations, consumers often experience lower satisfaction [35], envy [36], and schadenfreude [37].

Within the higher education setting, students often engage in social comparisons across various dimensions, including tuition costs—one of their largest expenditures—particularly among universities they know or consider peers. If a student’s own university keeps its tuition and fees constant over two years, while a counterpart university increases its tuition and fees in the second year (vs. also keeps its tuition and fees constant), the student may become relatively more satisfied with the price they pay. The rationale behind this is that, assuming the quality of education remains constant across the two years, students will perceive the value derived from their payments to be relatively higher compared to the previous year, due to social comparisons [38,39]. In other words, imagine a student’s university charges $20,000 across two years, while a counterpart university charges $25,000 the first year and $30,000 the second year. The value the student is obtaining in the second year relative to students at the counterpart university is better than the value the student obtained in the first year relative to students at the counterpart university. Consequently, the student should become more satisfied. In sum, we predict the following:

H2: Students perceive the price they pay for their tuition and fees to be of higher relative value when they learn that their tuition and fees remain constant, but a counterpart university increases its tuition and fees (vs. keeps these costs constant).

H3: The effect of condition (*Increase* vs. *Constant*) on students’ satisfaction with their overall university experience is serially mediated by relative value perceptions leading to satisfaction with tuition and fees.

## The present research

Across two studies, this research (materials and data are available here) investigated university students’ satisfaction with tuition and fees, and overall university experience. This work examined how their satisfaction changed between two years, across two conditions (*Increase* vs. *Constant*). In both conditions, the cost of tuition and fees for the participant’s university did not change over the two years. However, the cost of tuition and fees for a counterpart university varied. In the *Increase* condition, it went up in the second year; in the *Constant* condition, it remained the same over the two years. Study 1 aimed to examine whether the cost increase for the other university would cause participants to feel more satisfied with the cost of tuition and fees charged by their own university (H1a). Study 2 expanded the scope by also examining satisfaction with overall university experience (H1b) and the relative value participants perceived from their payment (H2-H3). The cost of tuition and fees is something that university students care greatly about [2,18], so it is likely that participants were quite motivated to be accurate and thorough when responding. The West Virginia University Office of Human Protections approved Study 1, and The University of Texas at San Antonio Institutional Review Board approved Study 2. Participants provided written consent in the research.

### Study 1 method

At the beginning of the Fall 2021 semester, an invitation was emailed to undergraduate students at a large, public university to participate in a research study in exchange for a chance to win one of a few $25 Amazon gift cards. The survey was kept open for seven days (8/16/2021–8/22/2021), which proved to be a sufficient time frame to acquire a large sample (see below).

Participants answered three eligibility questions before the main study started. The first asked which of two universities they attended: theirs, or a counterpart university (the counterpart university was chosen because it came from the same athletic conference and was of similar size and academic prestige; comparability between the two universities on these characteristics was important to prevent the perception that higher fees meant higher quality [40]). Those who answered incorrectly were deemed ineligible because they clearly were not paying attention. The second asked how many years they had left as an undergraduate student at their university after the Spring 2022 semester. Those with less than a year left were deemed ineligible because part of the study would ask them about the cost of tuition and fees during the following year. The third question asked whether they were full-time or part-time students. Part-time students were deemed ineligible because the remainder of the study was about the cost of tuition and fees for full-time students. In addition to answering the three eligibility questions, participants answered a question that asked whether they were in-state or out-of-state students. The remainder of the study was adjusted accordingly based on their response to this question. Participants were randomly assigned, with equal allocation, to the *Increase* or *Control* condition through simple randomization implemented in Qualtrics. The following sections outline the experimental procedure, first for in-state students, followed by the procedure for out-of-state students.

In-state students were presented with the (true) cost of tuition and fees for full-time, in-state undergraduate students at their university and the counterpart university for the upcoming year (i.e., Fall 2021 and Spring 2022). Their university would charge $9,144, whereas the other university would charge $13,280. Participants then responded to three items aimed at capturing their satisfaction with the 2021–2022 cost of tuition and fees at their university, using seven-point scales: i) How satisfied are you with the amount you have to pay in tuition and fees for the 2021–2022 academic year? (*1 = Not at all*, *7 = To a great extent*); ii) How pleased are you with the amount you have to pay in tuition and fees for the 2021–2022 academic year? (*1 = Not at all*, *7 = To a great extent*); iii) How would you describe your overall attitude toward the amount you have to pay in tuition and fees for the 2021–2022 academic year? (*1 = Extremely unfavorable*, 7 = *Extremely favorable*). Participants’ responses to these three items were averaged to form a single “2021 cost of attendance satisfaction index” (α = .94).

These participants were then presented with both universities’ (ostensible) tuition and fee plans for the following year (i.e., Fall 2022 and Spring 2023). The planned cost of tuition and fees at their university was said to be the same as before ($9,144). However, the planned cost of tuition and fees at the other university varied between-subjects. In the *Increase* condition, the other university was said to be planning to charge $15,280, whereas in the *Constant* condition, the other university was said to be planning to charge the same as before ($13,280). Participants then completed the same three satisfaction measures from earlier, only for 2022–2023. Their responses to these measures were averaged to form a single “2022 cost of attendance satisfaction index” (α = .96). The research focus was on the change in participants’ satisfaction with their cost of attendance across the two years. Thus, to create the dependent variable, change in cost of attendance satisfaction, participants’ 2021 cost of attendance satisfaction index was subtracted from their 2022 cost of attendance satisfaction index.

For participants who were out-of-state students, the procedure was the same, except that the amounts were adjusted based on true differences between in-state and out-of-state tuition costs. In 2021–2022 and 2022–2023, the cost of tuition and fees at their own university was $25,824. In 2021–2022, the cost of tuition and fees at the other university was $28,800. In 2022–2023, the cost of tuition and fees at the other university varied between subjects so that it was either $30,800 (*Increase*) or $28,800 (*Constant*).

At the end of the study, participants first answered an attention check question. The question asked whether their university and the other university planned to increase the 2022–2023 tuition and fees from 2021–2022. Those who answered incorrectly were excluded from analyses. Following this, participants responded to demographic questions, including their scholarship status, number of years as a student, student ID number (for gift card distribution purposes), age, gender, and email address (to contact in the event they won a gift card). Additionally, participants were probed for their thoughts about the study and its purpose. None of the participants questioned the information presented during the study, nor did they correctly guess the study’s purpose. About one month after the study, a debriefing email was sent to all participants, and gift cards were distributed to the lottery winners.

### Study 1 results and discussion

Altogether, Study 1 obtained responses from 748 eligible participants (640 after exclusions: 67% female; *M*_Age_ = 19.4, *SD*_Age_ = 2.1; 339 in-state participants, 301 out-of-state participants). A sensitivity power analysis was conducted to determine the smallest effect size detectable with the final sample. With *N* = 640, the study had 80% power (α = .05, two-tailed) to detect an effect of *f* = .11. Power was also examined for the in-state (*N* = 339) and out-of-state (*N* = 301) subsamples. The in-state subsample had 80% power to detect an effect of *f* = .15, and the out-of-state subsample had 80% power to detect an effect of *f* = .16. Thus, both the full sample and the subsamples were adequately powered to detect small-to-moderate effects [41,42].

**Fig 1:**
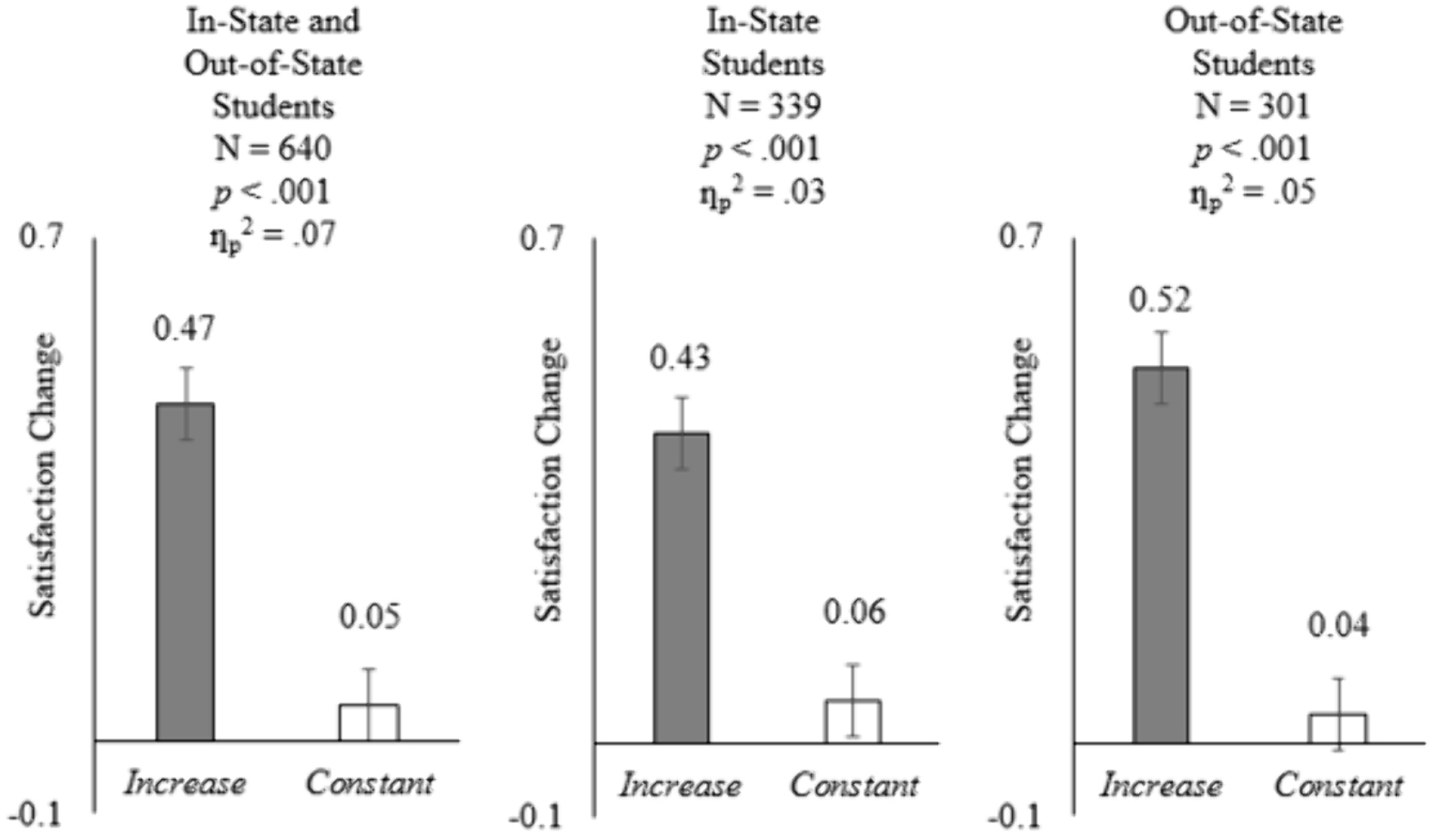
Comparisons between the two experimental conditions for Study 1. The bars depict the means, and the error bars represent the standard errors of the means. Left panel: overall sample, including both in-state and out-of-state students. Middle panel: in-state students. Right panel: out-of-state students.

ANOVA analysis showed that participants became more satisfied with the cost of attendance in the *Increase* (vs. *Constant*) condition (*M*_*Increase*_ = +.47, *SD*_*Increase*_ = .90 vs. *M*_*Constant*_ = +.05, *SD*_*Constant*_ = .53; *F*(1, 638) = 51.61, *p* < .001, ƞ_p_^2^ = .07; see Fig 1 left panel), consistent with H1a. Moreover, note that the results were similar for in-state (*M*_*Increase*_ = +.43, *SD*_*Increase*_ = .91 vs. *M*_*Constant*_ = +.06, *SD*_*Constant*_ = .52; *F*(1, 636) = 20.81, *p* < .001, ƞ_p_^2^ = .03; see Fig 1 middle panel) and out-of-state (*M*_*Increase*_ = +.52, *SD*_*Increase*_ = .89 vs. *M*_*Constant*_ = +.04, *SD*_*Constant*_ = .54; *F*(1, 636) = 31.85, *p* < .001, ƞ_p_^2^ = .05; see Fig 1 right panel) students. The effect sizes of these three pairs of comparisons fall in the small-to-medium range by conventional benchmarks, yet they are practically meaningful in consumer research [43]. The partial eta-squared values, ranging from.03 to.07, indicate that whether participants learned that a peer university increased its tuition and fees or not accounted for 3% to 7% of the variance in satisfaction changes between conditions—noteworthy proportions in behavioral research. The differences amounted to around half a scale point in satisfaction ratings, indicating that even a brief communication about peer universities’ tuition policies can noticeably influence how students evaluate their own university’s costs.

Overall, these findings demonstrate that students become relatively more satisfied with their cost of attendance when a counterpart university increases its cost of tuition and fees (vs. keeps the cost the same), even if their own tuition and fees remain unchanged. While Study 1 focused on investigating satisfaction with tuition and fees, Study 2 broadened the scope by examining satisfaction with students’ overall university experience, in addition to tuition and fees, while delving into the underlying psychological mechanism of relative value considerations.

### Study 2 method

Two hundred and two Prolific participants who were college students (166 after exclusions: 46% female; *M*_Age_ = 30.9, *SD*_Age_ = 10.4) completed the study in December 2024. The study employed two conditions (*Increase* vs. *Constant*). Participants were randomly assigned, with equal allocation, to the *Increase* or *Control* condition through simple randomization implemented in Qualtrics. Participants imagined that they were full-time undergraduate students at Summit University (SU) with a couple more years of school remaining. They were informed that SU was often compared to Horizon University (HU), which was similar to SU in terms of size, ranking, and campus life. As mentioned, comparability between the two universities on these characteristics was important to prevent the perception that higher fees meant higher quality [40]).

After reading the costs of tuition and fees for the 2024–2025 academic year for the two universities—SU charged $10,104 and HU $10,250—participants responded to three sets of measures. The first set assessed satisfaction with their tuition and fees using the three measures previously employed in Study 1. Participants’ responses to these three measures were averaged to form a single “2024 cost of attendance satisfaction index” (α = .94). The second set assessed their satisfaction with the overall university experience using the following items: i) How satisfied would you be as a student at SU during the 2024–2025 academic year? (*1 = Not at all*, *7 = To a great extent*); ii) How pleased would you be as a student at SU during the 2024–2025 academic year? (*1 = Not at all*, *7 = To a great extent*); iii) How would you describe your overall attitude toward SU as a student during the 2024–2025 academic year? (*1 = Extremely unfavorable*, 7 = *Extremely favorable*). Participants’ responses to these three items were averaged to form a single “2024 university experience satisfaction index” (α = .95). The third set asked for participants’ agreement or disagreement with the following two statements (*1 = Strongly disagree*, *7 = Strongly agree*): i) For the 2024–2025 academic year, I would feel like I am getting a good deal in terms of tuition and fees relative to the students at HU; ii) For the 2024–2025 academic year, I would think my tuition and fees offer better value compared to those at HU. Participants’ responses to these two items were averaged to form a single “2024 relative value index” (*r* = .84).

Participants were then presented with both universities’ tuition and fee plans for the 2025–2026 academic year. The planned cost of tuition and fees at their university was said to be the same as before ($10,104). However, the planned cost of tuition and fees at HU varied between-subjects. In the *Increase* condition, HU was said to be planning to charge $12,250, whereas in the *Constant* condition, HU was said to be planning to charge the same as before ($10,250). Participants then completed the same three sets of measures as described earlier, only for 2025–2026. Their responses formed the “2025 cost of attendance satisfaction index” (α = .95), “2025 university experience satisfaction index” (α = .95), and “2025 relative value index” (*r* = .89), respectively. The differences between the responses across the two academic years’ indices formed three dependent variables: change in cost of attendance satisfaction, change in university experience satisfaction, and change in relative value. At the end of the study, participants answered an attention check question (similar to that used in Study 1) and reported their age and gender.

### Study 2 results and discussion

A sensitivity power analysis was conducted for Study 2. With *N* = 166, the study had 80% power (α = .05, two-tailed) to detect an effect size of *f* = .22 across the main dependent variables (cost of attendance satisfaction, university experience satisfaction, and relative value). Accordingly, this sample size provided adequate power to detect effects in the small-to-moderate range.

**Fig 2:**
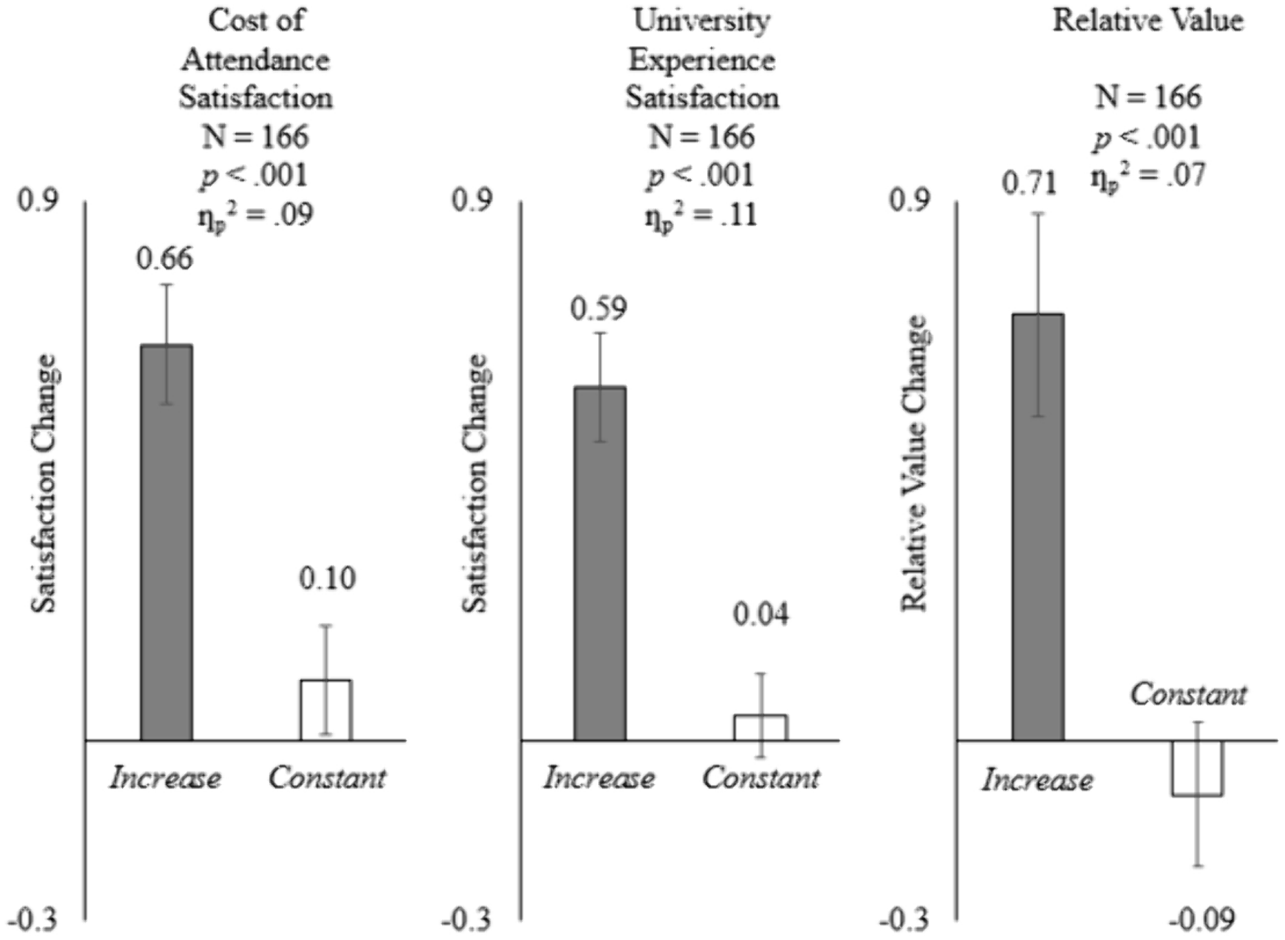
Comparisons between the two experimental conditions for Study 2. The bars depict the means and the error bars represent the standard errors of the means. Left panel: cost of attendance satisfaction. Middle panel: university experience satisfaction. Right panel: relative value.

ANOVA analysis showed that participants became more satisfied with the cost of attendance in the *Increase* (vs. *Constant*) condition (*M*_*Increase*_ = +.66, *SD*_*Increase*_ = 1.00 vs. *M*_*Constant*_ = +.10, *SD*_*Constant*_ = .75; *F*(1, 164) = 15.87, *p* < .001, ƞ_p_^2^ = .09; see Fig 2 left panel), providing support for H1a. Participants also became more satisfied with their university experience in the *Increase* (vs. *Constant*) condition (*M*_*Increase*_ = +.59, *SD*_*Increase*_ = .87 vs. *M*_*Constant*_ = +.04, *SD*_*Constant*_ = .61; *F*(1, 164) = 20.72, *p* < .001, ƞ_p_^2^ = .11; see Fig 2 middle panel), providing support for H1b. In addition, in line with H2, participants perceived the relative value as higher in the *Increase* (vs. *Constant*) condition (*M*_*Increase*_ = +.71, *SD*_*Increase*_ = 1.66 vs. *M*_*Constant*_ = −.09, *SD*_*Constant*_ = .98; *F*(1, 164) = 13.14, *p* < .001, ƞ_p_^2^ = .07; see Fig 2 right panel). The effect sizes of these three pairs of comparisons are considered medium to large, and practically meaningful, in consumer research [43]. The partial eta-squared values, ranging from.07 to.11, suggest that the *Increase* versus *Constant* manipulation explained a sizable 7% to 11% of the variance in satisfaction changes between conditions. These effects translated into changes of more than half a scale point in reported satisfaction, implying that tuition communications emphasizing relative value can meaningfully enhance students’ satisfaction with both their cost of attendance and their broader university experience.

**Fig 3:**
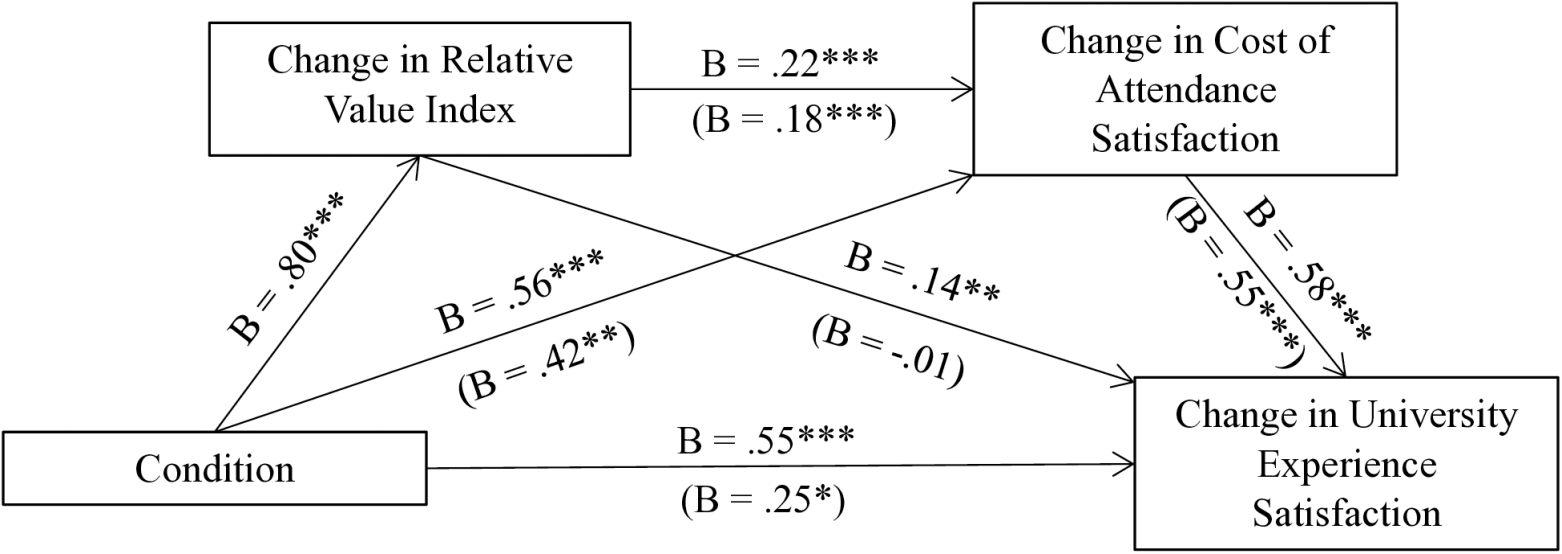
Study 2 serial mediation analysis (PROCESS Model 6). Condition coded as 0 = *Constant*, 1 = *Increase*. Coefficients in the parentheses are generated from a single regression when all independent variables are included. * *p* < .05. ** *p* < .01. *** *p* < .001.

A mediation analysis was run with 5,000 bootstrapped samples [44]. It employed PROCESS Model 6 to test whether the change in relative value perceptions and the change in cost of attendance satisfaction serially mediated the relationship between Condition (coded as *0* = *Constant*, *1* = *Increase*) and the change in university experience satisfaction (see Fig 3 for full mediation results). The 95% confidence interval of the bootstrapped indirect effect for this serial mediation did not include zero [.02,.20], indicating a significant indirect effect, in agreement with H3. This suggests that relative value considerations drove participants’ satisfaction with cost of attendance, which consequently drove their satisfaction with university experience.

In sum, Study 2 provided support for H1a-H3. Students became more satisfied with their cost of attendance and university experience when another university raised its tuition and fees. In addition, relative value considerations drove the greater satisfaction with tuition and fees, which led to greater satisfaction with overall university experience.

## General discussion

### Theoretical contributions and practical implications

This research demonstrates that students become relatively more satisfied when their own cost of tuition and fees remains constant but another university increases its cost of tuition and fees (vs. keeps these constant). This research offers theoretical and practical contributions. Theoretically, it shows that even if the price they pay does not change, consumers become relatively more satisfied when their peers’ costs increase (vs. do not change). This is consistent with the notion that consumers derive satisfaction from experiencing an increase in relative value compared to their peers over two periods [39]. Even though the price consumers are paying and the product they are receiving is held constant, consumers become more satisfied when they become aware that others have had their prices increased (vs. held constant). In sum, this work adds to the vast literature on social comparisons in consumption [33,34] by documenting the important role it plays in the crucial arena of university students’ satisfaction with higher education.

This research is practically important for many reasons. First, it highlights the important role that communicating relative value can play in increasing consumer satisfaction. Consistent with prior work [39], the present research demonstrates that consumers get satisfaction from perceiving an increase in the value of the deals they are getting relative to their peers. Therefore, universities with an advantage in their cost of attendance (i.e., universities that charge less) will benefit from highlighting comparisons to counterpart universities. Such comparisons underscore the good return for students’ investments and increase their satisfaction. Second, while increasing consumer satisfaction typically requires additional financial or time investments from organizations, this research demonstrates that this can be achieved without such investments. In other words, this work identifies a cost-free strategy through which organizations can increase consumer satisfaction. When employed responsibly, such communications offer students meaningful information, thereby promoting transparency and empowering informed decision-making. Third, whereas previous literature has primarily investigated consumers’ satisfaction with the products or services they receive (i.e., the benefits customers obtain [45–47]), this research investigates this aspect plus consumers’ satisfaction with what they are paying (i.e., the monetary cost customers incur [26,48]). The present work demonstrates that communicating that consumers’ costs compare favorably to what their peers are paying leads to increased satisfaction. Therefore, directing consumers’ attention to such monetary cost has the potential to significantly enhance their satisfaction. This is especially relevant in the higher education arena, given the current market factors (discussed earlier) that have had a major impact on many universities’ tuition prices and fees.

### Limitations and future research

This research is not without limitations. First, in both studies, a participant’s tuition was lower than that of the comparison university, leaving open the question of whether similar results would emerge if participants’ tuition began at the same level or higher than that of the counterpart university. Along these same lines, future research could test scenarios in which students experience tuition increases at their own university that are smaller than those at peer universities, rather than scenarios where their own tuition is held constant. Second, caution is warranted when generalizing the finding that relative tuition information shapes students’ overall university experience satisfaction. In Study 2, participants responded to a hypothetical scenario in which tuition was the only differentiating information provided about the two universities. This design was useful for isolating the effect of tuition, but it differs from real-world contexts where students have access to many other sources of information (e.g., academic quality, campus life, or reputation). In such cases, the influence of relative tuition information on overall experience satisfaction may be attenuated. Third, this work did not explore possible moderating factors. One such moderator could be the perceived gap in reputation or academic quality between the two universities used for comparison [18]. In this research, universities of similar standing were used in the studies, making them comparable. However, when the gap in reputation between two universities is large, students may perceive the schools as incomparable. Another potential moderator is culture. Participants in this research were from a Western, individualistic society [49]. In collectivistic societies, individuals may view themselves and their counterparts at a comparable university as part of a single collective, thus dampening the observed effects. In addition, this research did not address whether the observed effects on satisfaction are temporary or long-lasting. While the studies focused on short-term effects (measured immediately after participants learned about the costs of attendance), the question of whether the satisfaction boost persists over several years is an intriguing avenue for future research. Also, the satisfaction measurements dealt with students’ reactions to tuition costs and overall university experience, while leaving out an investigation into some other potential dependent measures (e.g., social life satisfaction, perception of the university’s academic reputation). These other aspects were not measured because they are not directly relevant to tuition and fees (albeit they might be indirectly connected). Broadening the scope of the satisfaction measurements to include additional aspects would allow for a more comprehensive understanding of the factors influencing student satisfaction.

## Supporting information

S1 File Appendix 1: Public universities’ costs of attendance published by the State University System of Florida. (DOCX)
