# Supplementary material for: Enhancing student satisfaction via social comparisons
Source: PLoS One. 2025 Nov 7;20(11):e0335910. doi: 10.1371/journal.pone.0335910 (PMC12594366; doi:10.1371/journal.pone.0335910)

Appendix 1: Public universities’ costs of attendance published by the State University System of Florida


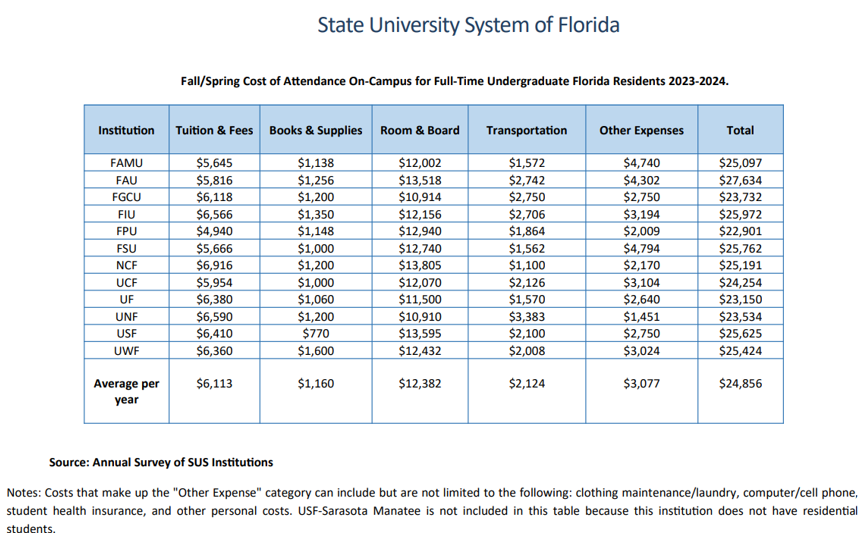

Supplement: S1 File — (DOCX) [file pone.0335910.s001.docx]
